# Bisulfite-based epityping on pooled genomic DNA provides an accurate estimate of average group DNA methylation

**DOI:** 10.1186/1756-8935-2-3

**Published:** 2009-03-10

**Authors:** Sophia J Docherty, Oliver SP Davis, Claire MA Haworth, Robert Plomin, Jonathan Mill

**Affiliations:** 1 Social Genetic and Developmental Psychiatry Centre, Institute of Psychiatry, King's College London, De Crespigny Park, Denmark Hill, London, SE5 8AF, UK

## Abstract

**Background:**

DNA methylation plays a vital role in normal cellular function, with aberrant methylation signatures being implicated in a growing number of human pathologies and complex human traits. Methods based on the modification of genomic DNA with sodium bisulfite are considered the 'gold-standard' for DNA methylation profiling on genomic DNA; however, they require relatively large amounts of DNA and may be prohibitively expensive when used on the large sample sizes necessary to detect small effects. We propose that a high-throughput DNA pooling approach will facilitate the use of emerging methylomic profiling techniques in large samples.

**Results:**

Compared with data generated from 89 individual samples, our analysis of 205 CpG sites spanning nine independent regions of the genome demonstrates that DNA pools can be used to provide an accurate and reliable quantitative estimate of average group DNA methylation. Comparison of data generated from the pooled DNA samples with results averaged across the individual samples comprising each pool revealed highly significant correlations for individual CpG sites across all nine regions, with an average overall correlation across all regions and pools of 0.95 (95% bootstrapped confidence intervals: 0.94 to 0.96).

**Conclusion:**

In this study we demonstrate the validity of using pooled DNA samples to accurately assess group DNA methylation averages. Such an approach can be readily applied to the assessment of disease phenotypes reducing the time, cost and amount of DNA starting material required for large-scale epigenetic analyses.

## Background

Epigenetics refers to the reversible regulation of various genomic functions mediated through partially stable modifications of DNA and chromatin histones. Epigenetic processes are essential for normal cellular development and differentiation, and allow the regulation of gene function through non-mutagenic mechanisms. Of particular interest is the phenomenon of cytosine methylation, occurring at position 5 of the cytosine pyrimidine ring in CpG dinucleotides. This process is intrinsically linked to the regulation of gene expression, with many genes demonstrating an inverse correlation between the degree of DNA methylation and the level of expression [1]. The methylation of these CpG sites, over-represented in CpG islands in the promoter regulatory regions of many genes, disrupts the binding of transcription factors and attracts methyl-binding proteins that are associated with gene silencing and chromatin compaction. DNA methylation plays a vital role in normal cellular function, and aberrant methylation signatures have thus been implicated in a growing number of human pathologies [2,3] including cancer [4], imprinting disorders [5], and even complex neuropsychiatric phenotypes such as schizophrenia and bipolar disorder [6]. The 'gold standard' method for mapping methylated cytosines is via the treatment of genomic DNA with sodium bisulfite; this process converts unmethylated cytosines to uracils (and subsequently, via PCR, to thymidines), while methylated cytosines are resistant to bisulfite and remain unchanged [7]. After sodium bisulfite treatment, DNA regions of interest are amplified and interrogated to identify C → T transitions or stable C positions, respectively corresponding to unmethylated and methylated cytosines in the native DNA. Numerous methods of analyzing bisulfite-modified DNA have been described [8], including methods based on the sequencing of bisulfite PCR amplicons (to obtain a strand-specific average) or the sequencing of cloned amplicons (to provide methylation maps of single DNA molecules).

Recently, several high-throughput methodologies have been developed to determine DNA methylation patterns from bisulfite-converted DNA templates including base-specific cleavage followed by MALDI-TOF mass spectrometry [9], and the use of next-generation deep-sequencing methodologies to enable the highly parallel analysis of bisulfite-treated samples [10]. Such highly quantitative DNA methylation analyses are clearly vital to our understanding of gene function and the role of epigenetic dysfunction in disease, but wisdom gained following recent large-scale genetic association studies suggests that extremely large sample sizes may be crucial in detecting the small effects expected in the highly complex disorders that contribute most to the global burden of disease [11]. The expense of such large-scale research remains prohibitive to many researchers, and this economic obstacle is bolstered further by the relatively large quantities of DNA required for bisulfite treatment, especially if multi-locus or whole-genome approaches are to be utilized, and by the fact that quantitative DNA methylation assessment, unlike genotypic assessment, requires technical replicates to ensure accuracy. Whilst the systematic assessment of DNA methylation has the potential to revolutionize our knowledge about the etiology of many complex disorders, current methods remain unsuitable for profiling the large sample cohorts likely to be required to detect pathogenic epimutations, especially for complex disorders or where multiple tissue types need to be assessed.

Validated pooling techniques are widely employed to increase throughput in studies of DNA sequence variation [12,13] and gene expression [14], and have allowed researchers to assess samples of sizes which would otherwise be economically infeasible. To date, however, few studies have systematically analyzed the applicability of DNA pooling for the analysis of DNA methylation. Dejeux and colleagues successfully used pyrosequencing to screen DNA methylation across five loci in pooled DNA samples [15]. However, by pooling samples subsequent to sodium bisulfite treatment, their approach is potentially affected by differential bisulfite conversion biases, and requires relatively large amounts of starting material from each sample. Furthermore, the accuracy of their pooling approach was only tested in pools comprising relatively small numbers of samples, although it is likely that much larger sample sizes will be required in etiological studies of complex disease phenotypes. We propose that a high-throughput DNA pooling approach would permit many more researchers to move into epigenetic analysis, and facilitate the use of emerging methylomic profiling techniques in large samples.

In this study we quantitatively assessed DNA methylation levels at 205 CpG sites across nine independent genomic regions in four DNA pools and for each of the 89 individual samples included in the pools, to provide the first systematic investigation of the utility of DNA pooling for bisulfite-based DNA methylation profiling experiments.

## Methods

### DNA pool construction

We obtained 89 high-quality Centre de'Etude du Polymorphism Humain (CEPH) genomic DNA samples extracted from transformed lymphoblastoid cell lines (Coriell Institute for Medical Research, NJ, USA). All samples were tested for degradation and quantified in triplicate using fluorimetry, employing PicoGreen^® ^dsDNA quantitation reagent (Cambridge Bioscience, UK). Aliquots of each sample were diluted 1:5 with TE buffer (10 mM Tris, 1 mM EDTA) to a working concentration of 50 ng/μl. Four DNA pools were constructed in total. Three independent pools were formed from the DNA of CEPH: 1) 'Mothers' (*N *= 29) 2) 'Fathers' (*N *= 30) and 3) 'Offspring' (*N *= 30) by combining equimolar amounts of DNA from each individual (300 ng; 6 ul at 50 ng/μl). A fourth 'Full' pool (*N *= 89) was formed by combining equimolar amounts of each of the Mothers, Fathers and Offspring DNA pools so that each individual sample contributed 150 ng to the final pool; this equated to combining 87 μl of Mothers pool with 90 μl from the Fathers pool and 90 μl from the Offspring pool.

### Genomic region selection

In order to investigate DNA pool performance, it was necessary to decide upon genomic regions for DNA methylation analysis. To acheive a thorough assessment of the accuracy of bisulfite-based profiling on pooled DNA samples it was desirable that we selected regions demonstrating considerable between-individual variation spanning a range of DNA methylation levels from unmethylated to fully methylated. Therefore we selected six regions nominated from ongoing studies in our laboratory (associated with the genes *DRD4, DAT1, ESR1, NR3C1, IGF2*, and *SERT*) that display high between-individual variability. As well as inter-individual variation however, it was important to select regions displaying variation in group averages when calculated from individual results, which between-DNA pool comparison might serve to reflect. With this in mind we selected three regions from the X chromosome (associated with the genes *AR*, *FMR1 *and *MAOA*). Assays were designed for these nine regions using the online Sequenom EpiDesigner software , and oligo sequences are given in Additional file 1.

### DNA methylation analysis

Sodium bisulfite treatment was performed on 375 ng of each individual sample and pool using the EZ-96 DNA Methylation Kit (Zymo Research, CA, USA) following the manufacturers' standard protocol. Bisulfite-PCR amplification was conducted using Hot Star *Taq *DNA polymerase (Qiagen, UK) and cycling conditions of 55 cycles with an annealing temperature of 57°C for *AR*, and 45 cycles with an annealing temperature of 56°C for all other amplicions (see Additional file 1 for additional details about the nine amplicons). DNA methylation analysis was conducted following bisulfite-PCR amplification using the Sequenom EpiTYPER system (Sequenom Inc, CA, USA) as described previously [16]. This technique employs base-specific cleavage followed by MALDI-TOF mass spectrometry in which the size ratio of the cleaved products provides quantitative methylation estimates for CpG sites within a target region [9]. The entire experiment, from sodium bisulfite-treatment onwards, was subsequently repeated in duplicate to control for technical variation, and to assess the reliability of the data produced.

### Statistical analysis

The accuracy of DNA methylation estimates generated from pooled DNA was assessed via Pearson's product-moment correlations with data averaged across the individual samples comprising each pool. As some CpG sites located within the same genomic region are in close physical proximity to each other, their DNA methylation levels are unlikely to be entirely independent. We therefore fit a linear mixed effects model to account for any possible influence of such spatial correlations between CpG sites, and implemented a bootstrapping technique – using sampling with replacement – to estimate confidence intervals on correlation coefficients.

## Results and discussion

**Figure 1:**
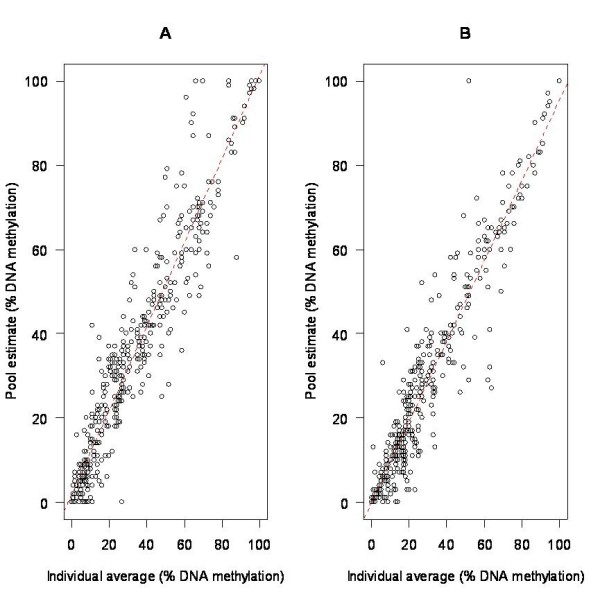
**Comparison of group average DNA methylation estimates obtained from pooled and individual DNA samples across 9 genomic regions**. DNA methylation estimates across the 205 CpG sites obtained from pooled DNA samples are highly correlated with actual average DNA values obtained from individual DNA samples in both A) the first and B) the second technical replicates of this experiment.

In total we assessed DNA methylation levels at 205 CpG sites – assessed by the Sequenom EpiTYPER platform as 133 CpG units – across nine independent genomic regions in each of the 89 CEPH individuals and four DNA pools, with all analyses being performed in duplicate. Comparison of data generated from the pooled DNA samples with results averaged across the individual samples comprising each pool revealed highly significant correlations (*P *< 2.2*10^-16^) for individual CpG sites across all nine regions (see Table 1), which remained when a linear mixed effects model was used to account for the spatial correlation between CpG sites within each amplicon. The overall correlation across all CpG sites assessed was 0.95 (95% bootstrapped confidence intervals: 0.94 to 0.96) in the first replicate (see Figure 1A) and 0.95 (95% bootstrapped confidence intervals: 0.93 to 0.96) in the second replicate (see Figure 1B), with an overall correlation of 0.95 (95% bootstrapped confidence intervals: 0.94 to 0.96) across the averaged data from both replicates. This correlation is comparable to the correlation of 0.95 seen between technical replicates, that is, the results gathered from the same individual samples in the first and second replicates. Our analyses yielded similar results when each of the DNA pools was assessed separately, demonstrating no effect of overlap between individuals within each pool on the overall correlation (see Table 2). Whilst virtually all the pooled DNA methylation estimates correlated very strongly with the group averages ascertained from profiling individual samples, the overall performance of some amplicons was better than others, suggesting that assay design may be important. The lowest correlations are seen in *ESR1 *– which may be explained by the fact that the first round of reactions failed for this amplicon, so the estimate is based on only one replicate. A linear mixed effects model showed that pool type had no significant effect on the overall correlations for a region. Furthermore, a range of pool sizes were employed here to assist in determining an ideal pool size for DNA methylation estimation. With little difference in performance demonstrated (see Table 2) it would appear that pools of up to 89 individuals perform to the same high standard.

**Table 1:** Correlations between pooled DNA methylation estimates and group averages assessed by individual sample analysis for CpG sites in each of the 9 genomic regions studied.

	***FMR1***	***DRD4***	***SERT***	***IGF2***	***GR***	***ESR1***	***DAT1***	***MAOA***	***AR***	**ALL**
**Individual average vs pool estimate – replicate 1**	0.95	0.93	0.93	0.93	0.98	-	0.95	0.89	0.94	0.95
**Individual average vs pool estimate – replicate 2**	0.81	0.97	0.88	0.95	0.90	0.81	0.92	0.72	1.00	0.95
**Individual data – replicate 1 vs replicate 2**	0.83	0.97	0.93	0.95	0.87	-	0.94	0.93	0.94	0.95
**Individual average vs pool estimate – average across both replicates**	0.95	0.95	0.95	0.93	0.98	0.81	0.97	0.86	0.98	0.95

The overall correlation between the averaged individual data and the pooled estimate (0.95) is the same as that between replicates of the individual data (0.95).

**Table 2:** Correlations between the methylation estimates, averaged across two replicates, of four DNA pools and group averages assessed by individual sample analysis for CpG sites in each of the nine genomic regions studied.

**DNA pool**	***n***	***FMR1***	***DRD4***	***SERT***	***IGF2***	***GR***	***ESR1***	***DAT1***	***MAOA***	***AR***	**ALL**
**Mothers**	29	0.93	0.86	0.96	0.94	0.99	0.88	0.98	0.91	0.99	0.93

**Fathers**	30	0.99	0.98	0.94	0.95	1.00	0.62	0.95	0.72	0.98	0.96

**Offspring**	30	0.95	0.98	0.98	0.96	0.99	0.88	0.98	0.91	0.97	0.96

**Full**	89	0.95	0.99	0.96	0.92	1.00	0.96	0.99	0.85	0.98	0.95

**Figure 2:**
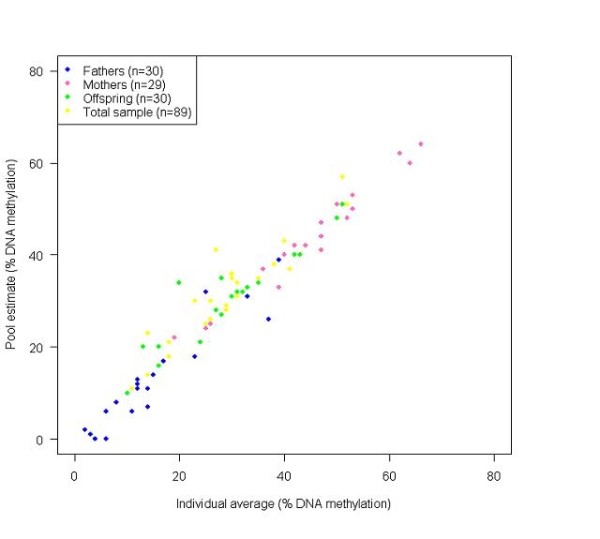
**Group average DNA methylation estimates from pooled and individual DNA samples for the androgen receptor (AR) amplicon on the X-chromosome**. Both the pool estimates and group averages based on individual DNA methylation data for the androgen receptor (*AR*) gene on the X chromosome clearly demonstrate the expected sex differences in DNA methylation with 'offspring' and 'total sample' pools (50% male, 50% female) showing intermediate levels of DNA methylation compared with the high level of methylation in the 'mothers' pool and low level of methylation in the 'fathers' pool.

**Figure 3:**
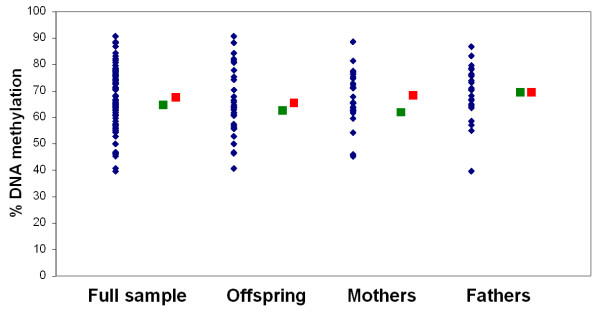
**Amplicon-averaged data from the most variably methylated autosomal region (*DRD4*)**. Blue diamonds denote DNA methylation for individual samples, green squares denote the pooled estimate, and red squares denote the average of the individual values for each group (total sample, offspring, mothers, and fathers). Even in this region, where individual DNA methylation values ranged from 40% to 91%, pooled estimates accurately predicted group averages.

**Figure 4:**
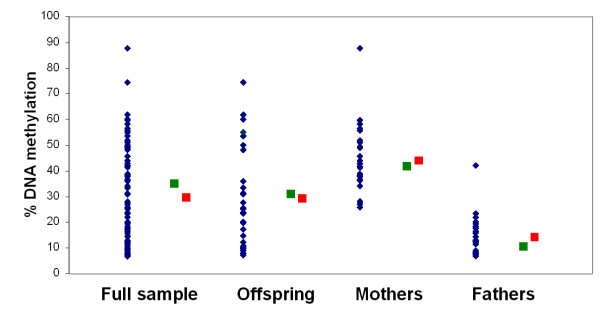
**Amplicon-averaged data from the most variably methylated X-chromosome region (*AR*)**. Blue diamonds denote DNA methylation for individual samples, green squares denote the pooled estimate, and red squares denote the average of the individual values for each group (total sample, offspring, mothers, and fathers). Even in this region, where individual DNA methylation values ranged from 7% to 88%, pooled estimates accurately predicted group averages.

For those regions located on the X chromosome, the DNA pool results clearly reflected the large sex differences in DNA methylation expected as a result of X-inactivation in females (Figure 2). Furthermore, the pooled DNA accurately estimated group averages across even those regions showing considerable between-individual variation (see Figures 3 and 4). Remarkably, the average absolute difference between the 'pooled' DNA methylation estimate and the 'real' average, determined by assessing individual samples, was 6.0% in the first set of experiments and 4.8% in the second set of replicates. This approximates to the normal level of between-replicate variability expected using the Sequenom EpiTYPER approach [9] and suggests that the accurate pooling of DNA prior to sodium bisulfite treatment does not introduce any significant error beyond that resulting from normal technical variability.

Our data indicate that the DNA methylation profiles obtained from pooled genomic DNA samples are remarkably consistent with those obtained from averaging the values for individual samples in a group, even in large pools of individual samples and for regions of the genome demonstrating high levels of between-individual DNA methylation variation. Obtaining methylation data from DNA pools could be extremely useful in instances where the availability of DNA from valuable sample collections is low, or where it is unfeasible to apply the desired profiling methodology to large numbers of samples. In such cases, it may be desirable to screen group averages using pooled DNA, in order to identify interesting regions warranting further investigation on an individual sample basis. While we assessed the validity of DNA pooling for DNA methylation profiling using the Sequenom EpiTYPER mass-spectrometry system, this method is potentially applicable to all bisulfite-based mapping techniques. Combined with microarrays and next-generation deep-sequencing technologies, for example, the bisulfite-based assessment of pooled genomic DNA should enable high-resolution methylation profiling to be performed across the large sample sizes required for detecting epimutations associated with pathological conditions. The utility of pooled genomic DNA in combination with high-resolution bisulfite mapping on next-generation sequencing platforms was highlighted by a recent study on pooled leukemia samples [10], and our data suggest that the conclusions from such studies are likely to be valid. This approach should facilitate the high-throughput assessment of disease phenotypes, reducing the time, cost and amount of DNA starting material required for large-scale epigenetic analyses.

## Conclusion

Compared with data generated from 89 individual samples, our analysis of 205 CpG sites spanning nine independent regions of the genome demonstrates that DNA pools can be used to provide an accurate and reliable quantitative estimate of average group DNA methylation using the Sequenom EpiTYPER system. Such an approach may be especially useful in highlighting regions of the genome for further analysis in large-scale epigenetic assessment of disease phenotypes – reducing the time, cost and amount of DNA starting material required.

## Competing interests

The authors declare that they have no competing interests.

## Authors' contributions

SJD performed the majority of the laboratory work and helped prepare the manuscript. CMAH, OSPD and RP helped conceive the study and provided statistical support. JM supervised the project and produced the final manuscript. All authors read and approved the final manuscript.

## Supplementary Material

Additional File 1 **Bisulfite-specific oligo primer sequences for the nine amplicons assessed in this study. **The number CpG units assayed in each amplicon are shown, with some units covering multiple CpG sites. Click here for file

## References

[B1] Jaenisch R, Bird A (2003). Epigenetic regulation of gene expression: how the genome integrates intrinsic and environmental signals. Nat Genet.

[B2] Hatchwell E, Greally JM (2007). The potential role of epigenomic dysregulation in complex human disease. Trends Genet.

[B3] Robertson KD (2005). DNA methylation and human disease. Nat Rev Genet.

[B4] Jones PA, Baylin SB (2007). The epigenomics of cancer. Cell.

[B5] Feinberg AP (2007). Phenotypic plasticity and the epigenetics of human disease. Nature.

[B6] Mill J, Tang T, Kaminsky Z, Khare T, Yazdanpanah S, Bouchard L, Jia P, Assadzadeh A, Flanagan J, Schumacher A, Wang SC, Petronis A (2008). Epigenomic profiling reveals DNA-methylation changes associated with major psychosis. Am J Hum Genet.

[B7] Clark SJ, Harrison J, Paul CL, Frommer M (1994). High sensitivity mapping of methylated cytosines. Nucleic Acids Res.

[B8] Clark SJ, Statham A, Stirzaker C, Molloy PL, Frommer M (2006). DNA methylation: bisulphite modification and analysis. Nat Protoc.

[B9] Ehrich M, Nelson MR, Stanssens P, Zabeau M, Liloglou T, Xinarianos G, Cantor CR, Field JK, Boom D van den (2005). Quantitative high-throughput analysis of DNA methylation patterns by base-specific cleavage and mass spectrometry. Proc Natl Acad Sci USA.

[B10] Taylor KH, Kramer RS, Davis JW, Guo J, Duff DJ, Xu D, Caldwell CW, Shi H (2007). Ultradeep bisulfite sequencing analysis of DNA methylation patterns in multiple gene promoters by 454 sequencing. Cancer Res.

[B11] (2007). Genome-wide association study of 14,000 cases of seven common diseases and 3,000 shared controls. Nature.

[B12] Docherty SJ, Butcher LM, Schalkwyk LC, Plomin R (2007). Applicability of DNA pools on 500 K SNP microarrays for cost-effective initial screens in genomewide association studies. BMC Genomics.

[B13] Kirov G, Nikolov I, Georgieva L, Moskvina V, Owen MJ, O'Donovan MC (2006). Pooled DNA genotyping on Affymetrix SNP genotyping arrays. BMC Genomics.

[B14] Kendziorski C, Irizarry RA, Chen KS, Haag JD, Gould MN (2005). On the utility of pooling biological samples in microarray experiments. Proc Natl Acad Sci USA.

[B15] Dejeux E, Audard V, Cavard C, Gut IG, Terris B, Tost J (2007). Rapid identification of promoter hypermethylation in hepatocellular carcinoma by pyrosequencing of etiologically homogeneous sample pools. J Mol Diagn.

[B16] Coolen MW, Statham AL, Gardiner-Garden M, Clark SJ (2007). Genomic profiling of CpG methylation and allelic specificity using quantitative high-throughput mass spectrometry: critical evaluation and improvements. Nucleic Acids Res.

