# Supplementary material for: Bisulfite-based epityping on pooled genomic DNA provides an accurate estimate of average group DNA methylation
Source: Epigenetics Chromatin. 2009 Mar 10;2:3. doi: 10.1186/1756-8935-2-3 (PMC2657899; doi:10.1186/1756-8935-2-3)
Supplement: Additional File 1 — Bisulfite-specific oligo primer sequences for the nine amplicons assessed in this study. The number CpG units assayed in each amplicon are shown, with some units covering multiple CpG sites. [file 1756-8935-2-3-S1.doc]

| **Gene** | **Genomic Region*** | **CpG units assessed** | **Primer Sequences**** |
| --- | --- | --- | --- |
| Androgen Receptor (*AR*) | chrX:66,680,331-66,680,666 | 11 CpG units (18 CpG sites) | F: TTTAAATTTTGGTTTAGGAAAGTAGGA  R: CCTCCTCTACCTCTAAACTTACTCC |
| Dopamine D4 Receptor (*DRD4*) | chr11:626,509-626,904 | 19 CpG units (32 CpG sites) | F: GGGATTTTTTGTTTAGGGTTAGAGG  R: CACCCTAATCCACCTAATATCTAACA |
| Dopamine Transporter (*DAT1*) | chr17:25,586,562-25,586,938 | 14 CpG units (23 CpG sites) | F: GGGTGGGTATTTGGTGTAGTTAT  R: AACTCTAACAAACAACTTTCCCTACA |
| Estrogen Receptor Alpha (*ESR1*) | chr6:152,171,138-152,170,750 | 18 CpG units (26 CpG sites) | F: TTAGGTAGTAGGGTATTTGTTGGT  R: AACCCTCCACACCAAAACATCTAAA |
| Fragile X Mental Retardation 1 (*FMR1*) | chrX:146,801,594-146,801,918 | 16 CpG units (26 CpG sites) | F: GATTTGGGGTTTGTTGGAAGT  R: CCAAAAAAAACCTAAAACCCTCTTA |
| Glucocorticoid Receptor (*NR3C1*) | chr5:142,763,696-142,764,098 | 13 CpG units (20 CpG sites) | F: TTTAATTTTTTAGGAAAAAGGGTGG  R: CCCTAAAACCTCCCCAAAAA |
| Insulin-like Growth Factor 2 (*IGF2*) | chr11:2,111,119-2,110,666 | 18 CpG units (23 CpG sites) | F: TATAGGGGTGGTTTGTTAGGTTAGG  R: AAATCAAAAAAAACCCCAAAAAAAC |
| Monamine Oxidase A (*MAOA*) | chrX:43,400,526-43,400,744 | 7 CpG units (8 CpG sites) | F: GTTAAAGTATGGAGAATTAAGAGAAGG  R: CAAAATATAAAACCAAACCATAACTACA |
| Serotonin Receptor (*SERT*) | chr17:25,586,562-25,586,938 | 17 CpG units (29 CpG sites) | F: GGTTAGTTTTAGTTTTGGTTTTTGTT  R: CAAAAATTCTTCAAAAACTCTTTAAC |

*UCSC March 2006 Genomic Assembly

**Primers were ordered with the standard Sequenom MassCLEAVE tails (F: aggaagagag, R: cagtaatacgactcactatagggagaaggct)
